# Efficacy of vitamin K2 in the prevention and treatment of postmenopausal osteoporosis: A systematic review and meta-analysis of randomized controlled trials

**DOI:** 10.3389/fpubh.2022.979649

**Published:** 2022-08-11

**Authors:** Ming-ling Ma, Zi-jian Ma, Yi-lang He, Hao Sun, Bin Yang, Bin-jia Ruan, Wan-da Zhan, Shi-xuan Li, Hui Dong, Yong-xiang Wang

**Affiliations:** ^1^Department of Orthopedics, Northern Jiangsu People's Hospital Affiliated to Yangzhou University/Clinical Medical College, Yangzhou University, Yangzhou, China; ^2^Department of Graduate School, Dalian Medical University, Dalian, China; ^3^Yangzhou University Medical College, Yangzhou, China

**Keywords:** vitamin K2, postmenopausal women, osteoporosis, fracture, meta-analysis, randomized controlled trial

## Abstract

**Introduction:**

Vitamin K (VK) as a nutrient, is a cofactor in the carboxylation of osteocalcin (OC), which can bind with hydroxyapatite to promote bone mineralization and increase bone strength. However, some studies have been inconsistent on whether vitamin K2 (VK2) can maintain or improve bone mineral density (BMD) and reduce the incidence of fractures in postmenopausal women. Therefore, the main objective of this meta-analysis was to determine the effect of VK2 as a nutritional supplement on BMD and fracture incidence in postmenopausal women.

**Methods:**

We searched PubMed, EMBASE, and Cochrane Library databases (published before March 17, 2022) and then extracted and pooled data from all randomized controlled trials (RCTs) that met the inclusion criteria.

**Results:**

Sixteen RCTs with a total of 6,425 subjects were included in this meta-analysis. The overall effect test of 10 studies showed a significant improvement in lumbar spine BMD (BMD LS) (*P* = 0.006) with VK2. The subgroup analysis of VK2 combination therapy showed that BMD LS was significantly maintained and improved with the administration of VK2 (*P* = 0.03). The overall effect test of the six RCTs showed no significant difference in fracture incidence between the two groups (RR=0.96, P=0.65). However, after excluding one heterogeneous study, the overall effect test showed a significant reduction in fracture incidence with VK2 (RR = 0.43, *P* = 0.01). In addition, this meta-analysis showed that VK2 reduced serum undercarboxylated osteocalcin (uc-OC) levels and the ratio of uc-OC to cOC in both subgroups of VK2 combined intervention and alone. However, for carboxylated osteocalcin (cOC), both subgroup analysis and overall effect test showed no significant effect of VK2 on it. And the pooled analysis of adverse reactions showed no significant difference between the VK2 and control groups (RR = 1.03, 95%CI 0.87 to 1.21, *P* = 0.76).

**Conclusions:**

The results of this meta-analysis seem to indicate that VK2 supplementation has a positive effect on the maintenance and improvement of BMD LS in postmenopausal women, and it can also reduce the fracture incidence, serum uc-OC levels and the ratio of uc-OC to cOC. In conclusion, VK2 can indirectly promote bone mineralization and increase bone strength.

## Introduction

Osteoporosis (OP) has become a global public health problem, which is mainly characterized by the reduction of bone trabecular number, destruction of bone microstructure and the increase of bone fragility, which predisposes to fractures (1). OP and OP-related fractures are increasingly common in women over 55 years and men over 65 years, especially in older postmenopausal women (2). Hip fracture due to OP in particular can be devastating to the elderly, because it is expensive to treat and carries a high risk of death (3). Postmenopausal osteoporosis (PMOP) is the most common type in primary osteoporosis. When a woman goes through menopause, a sharp drop of estrogen levels will lead to more osteoclastogenesis than osteoblastogenesis, which can lead to the increase of bone resorption, the breakdown of bone metabolism balance, and eventual OP.

Osteocalcin (OC), as a biomarker of bone metabolism, is synthesized and secreted by osteoblasts. It plays an important role in regulating bone calcium metabolism. However, undercarboxylated osteocalcin (uc-OC) has no biological function, because it cannot bind to hydroxyapatite and does not facilitate the deposition of bone calcium. Only with the assistance of VK can uc-OC be converted to carboxylated osteocalcin (cOC), which has a high affinity for calcium and promotes hydroxyapatite formation and bone mineralization (4–7). VK is a fat-soluble vitamin that is a cofactor for the carboxylation of osteocalcin (8, 9). VK includes phylloquinone (VK1) and menaquinone (VK2) (10, 11). VK1 is mainly found in plants, while VK2 is mainly found in some fermented foods, most commonly natto, and VK2 can also be synthesized by intestinal bacteria (12). In addition, there are twelve MK subtypes in VK2, among which MK-4 and MK-7 mainly maintain bone health, as MK-4 and MK-7 can promote bone calcium deposition and increase bone strength (13).

But so far, the answer to the question of whether VK2 significantly maintains or improves bone mineral density (BMD) in various parts of the body and whether it significantly reduces the incidence of fracture is unclear. Some studies (14–20) suggest that VK2 can do this, but some suggest that it cannot (9, 11, 21). However, unlike VK2, the role of vitamin D and calcium in the prevention and treatment of OP has been confirmed by high-quality studies and has been included in guidelines for the prevention and treatment of OP (22, 23). Previous studies have shown that VK2 can promote the mineralization of 1,25 (OH)_2_D_3_-induced in human osteoblasts, and that the effect of VK2 in combination with vitamin D3 or calcium or other anti-osteoporosis drugs (such as alendronate) on bone mass is much greater than that of VK2 alone (24–27). Although previous meta-analyses have evaluated the effects of VK (28–34), to our knowledge, no meta-analysis has examined the effects of VK2 combination therapy and VK2 alone therapy on PMOP. Therefore, we consider it necessary to conduct this systematic review and meta-analysis to determine the role of VK2 in the prevention and treatment of PMOP.

## Materials and methods

### Search strategy

According to the PRISMA statement, we searched those authoritative literature databases (PubMed, EMBASE, and the Cochrane Library). The search terms used were vitamin K, menaquinone, menatetrenone, postmenopausal and osteoporosis. In order to retrieve these studies comprehensively, we adopted the search method of combining keywords and Boolean Operators. Medical Subject Heading (MeSH) phrases were also used appropriately, and we did not limit the year and language of publication during the search. We have stored the search strategy for the above database in Supplementary material.

### Inclusion and exclusion criteria

Based on the principles of PICOS (Population/Intervention/ Comparison/Outcome/Study design), the following inclusion criteria were developed: 1. Study design was a randomized controlled trial; 2. Study population was postmenopausal women; 3. Intervention group was VK2 alone or in combination with other therapeutic measures; 4. Control group was given another drug or placebo; 5. Outcomes should include at least one of the following:(1) percentage change in BMD of lumbar spine, femoral neck, hip, or forearm; (2) Incidence of fractures; (3) percentage change in serum uc-OC; (4) percentage change in serum cOC; (5) percentage change in serum uc-OC to cOC ratio and (6) incidence of adverse reactions.

Studies would be excluded if they fulfilled any of the following conditions: 1. Non-randomized controlled trial; 2. Study was not about VK2; 3. Subjects had renal or immune disease; 4. Subjects were taking anti-osteoporosis drugs or other drugs that affect bone metabolism (such as glucocorticoids or immunosuppressants); 5. Subjects were taking drugs that affect the absorption or metabolism of VK2, such as warfarin.

### Study selection

In accordance with the inclusion and exclusion criteria, two reviewers (ML. MA, ZJ.MA) browsed through the titles and abstracts of all studies for initial screening. Then, the full text of the studies was read carefully to identify the final included studies. During the screening process, two reviewers documented the reasons for exclusion of some studies. When two reviewers disagreed on the inclusion or exclusion of studies, a third reviewer (H. DONG) would be invited to discuss and make a final decision.

### Data extraction

First, three reviewers (ML. MA, ZJ.MA and H.SUN) extracted and collated the basic information of the final included studies including author, publication year, study type, country, number of study center, sample size, age, follow-up time, intervening measures, as well as other relevant data required for this meta-analysis. Then, three reviewers assessed the quality of the included studies. Some studies presented results in bar or line graphs, without giving specific data. In view of this, we used the online tool for graphical data extraction (Web Plot Digitizer, https://automeris.io/WebPlotDigitizer/). The extracted and collated data were finally rechecked by ML.MA.

### Outcomes

The percentage change in BMD (lumbar spine, femoral neck, hip, and forearm) before and after treatment with VK2 or other measures and the incidence of fractures were taken as primary outcomes. Meanwhile, the percentage change of serum uc-OC, cOC, and uc-OC to cOC ratio and the incidence of adverse reactions were taken as secondary outcomes.

### Assessment of bias risk for included studies

Two reviewers (YL.HE, SX.LI) independently assessed the risk of bias for all included RCTs using the Cochrane Collaboration's tool. This tool assessed the risk of bias for RCTs through the following seven lists: (1) Random sequence generation (selection bias); (2) Allocation concealment (selection bias); (3) Blinding of participants and personnel (performance bias); (4) Blinding of outcome assessment (detection bias); (5) Incomplete outcome data (attrition bias); (6) Selective reporting (reporting bias); (7) Other bias. Each list has three options: high risk, low risk and unclear risk.

### Data synthesis and analysis

We used the STATA software (STATA Software Version 16.0) to assess publication bias and conduct sensitivity analysis, and used the Review Manager Software (RevMan5.3) to make analysis for other data. For continuous outcome variables, we extracted the mean and standard deviation from the included studies and then calculated the weighted mean difference (WMD) and 95% confidence interval (95% CI), and for binary outcome variables, we extracted the number of positive events in both groups then calculated the risk ratio (RR) and 95%CI. The heterogeneity of studies was assessed using *I*^2^ statistics and Cochran's Q Test (35–37). The random-effects model was used when the heterogeneity between studies was large (*P* ≤ 0.1, *I*^2^ > 50%) and the fixed-effects model was used when the heterogeneity between studies was small (*P* > 0.1, *I*^2^ ≤ 50%). Subgroup analysis was based on VK2 and the studies were then divided into two subgroups: VK2 combined intervention subgroup and VK2 alone intervention subgroup. Sensitivity analysis was performed by removing studies that caused heterogeneity. The continuous variables in this meta-analysis were the percentage change of outcomes before and after treatment, which contributed to the elimination of differences in baseline data.

## Results

### Search results

**Figure 1:**
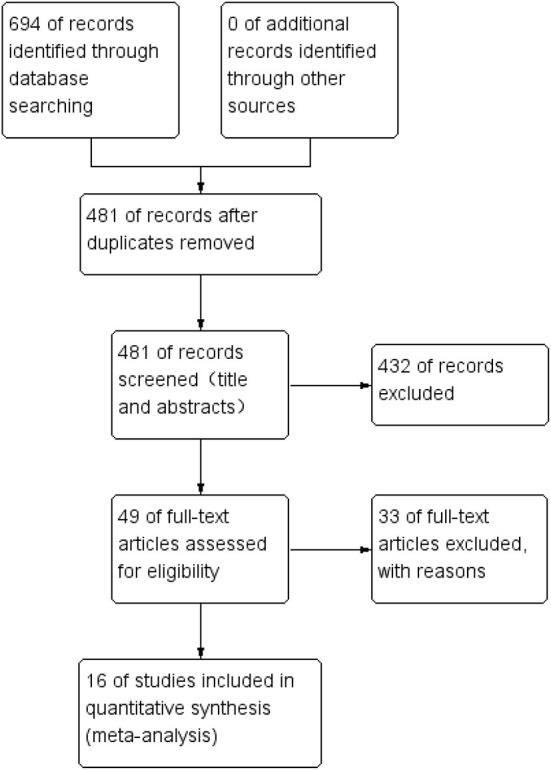
Flow diagram of studies selection.

We searched a total of 694 studies in PubMed, EMBASE and Cochrane Library databases, and then we removed 213 duplicated studies and obtained 481 studies initially. Next, by reading the titles and abstracts of these studies, we excluded 432 studies again for the following reasons:(1) subjects were uncorrelated with ours; (2) review or meta-analysis; (3) animal studies; (4) outcomes of studies did not meet our needs; (5) study population included either men or non-menopausal women; (6) mechanism researches for VK2. Next, the full texts of the remaining 49 studies were read precisely and carefully, and 16 studies (9, 14, 15, 18, 21, 26, 27, 38–46) were finally eligible for the inclusion in this meta-analysis. Another 33 studies were excluded for the following reasons: (1) the intervention was VK1 or partially VK1; (2) non-randomized controlled trials; (3) no access to study data; (4) study data were absolute values, not percentage change. Additionally, the majority of the included studies were conducted in Japan (18, 26, 27, 39–41, 44–46), and others were conducted in Denmark, the Netherlands, Norway, China and Indonesia (9, 14, 15, 21, 38, 42, 43). The screening process of the studies is shown in Figure 1.

### Characteristics of the included studies

Through searching and screening, 16 studies were included in this meta-analysis, all of which were RCTs with a total of 6,425 subjects. These studies were mainly distributed in Europe (9, 15, 21, 38, 42) and Asia (14, 18, 26, 27, 39–41, 43–46). Most of them were single-center studies, and only three were multicenter studies (14, 21, 41). The characteristics of the included studies are shown in Table 1.

**Table 1:** Characteristics of included studies.

**Reference**	**Study type**	**Country**	**Research center**	**Number**	**Age**	**Years of menopause**	**Osteoporosis**	**Follow-up**	**Intervention**	**Comparison**
Rønn et al. (9)	RCT	Denmark	SC	142	67.3 ± 4.4	>2	No	36 M	375μg/day MK-7 + 38μg/day VD3 + 800 mg/day Ca	38μg/day VD3 + 800 mg/day Ca
Rønn et al. (38)	RCT	Denmark	SC	142	60–80	>2	No	12 M	375μg/day MK-7 + 38μg/day VD3 + 800 mg/day Ca	38μg/day VD3 + 800 mg/day Ca
Kasukawa et al. (26)	RCT	Japan	SC	101	>60	-	Yes	12 M	17.5 mg/week risedronate + 45 mg/day menatetrenone	17.5 mg/week risedronate
Koitaya et al. (39)	RCT	Japan	SC	48	58.4 ± 3.8	7.3 ± 3.8	No	12 M	1.5 mg/day MK-4	placebo
Jiang et al. (14)	RCT	China	MC	213	45–75	>5	Yes	12 M	45 mg/day menatetrenone + 500 mg/day Ca	0.5 μg/day alfacalcidol + 500 mg/day Ca
Knapen et al. (15)	RCT	Netherlands	SC	244	55–65	9 ± 6	No	36 M	180μg/day MK-7	placebo
Emaus et al. (21)	RCT	Norway	MC	334	50–60	1-5	No	12 M	360 mg/day MK-7	placebo
Shiraki et al. (40)	RCT	Japan	SC	121	68.6 ± 7.6	19.6 ± 9.3	Yes	6 M	45 mg/day menatetrenone	133.8 mg/day Ca
Inoue et al. (41)	RCT	Japan	MC	4378	>50	20.55 ± 9.89	Yes	48 M	45 mg/day menatetrenone + Ca	Ca
Hirao et al. (27)	RCT	Japan	SC	48	68.5 ± 2.1	-	Yes	12 M	45 mg/day VK2 + 5 mg/day alendronate	5 mg/day alendronate
Knapen et al. (42)	RCT	Netherlands	SC	325	55–75	>2	No	36 M	45 mg/day MK-4	placebo
Purwosunu et al. (43)	RCT	Indonesia	SC	63	60–75	>2	Yes	12 M	45 mg/day menatetrenone + 1500 mg/day Ca	placebo + 1500 mg/day Ca
Ushiroyama et al. (44)	RCT	Japan	SC	172	53.41 ± 5.90	2.50 ± 4.16	Yes	24 M	45 mg/day MK-4 + 1μg/day VD3	1μg/day VD3
Iwamoto et al. (45)	RCT	Japan	SC	72	53-78	>5	Yes	24 M	45 mg/day MK-4	2 g/day Ca
Iwamoto et al. (46)	RCT	Japan	SC	92	55-81	>5	Yes	24 M	45 mg/day menatetrenone + 0.75 μg/day VD3	0.75 μg/day VD3
Iwamoto et al. (18)	RCT	Japan	SC	72	53.82 ± 5.24	5.21 ± 4.18	No	12 M	45 mg/day MK-4	1.0 g/day VD3

RCT, Randomized Controlled Trial; SC, single-center; MC, multiple-center; M, month; VK2, vitamin K2; VD3, vitamin D3; Ca, calcium.

### Quality assessment of the included studies

**Figure 2:**
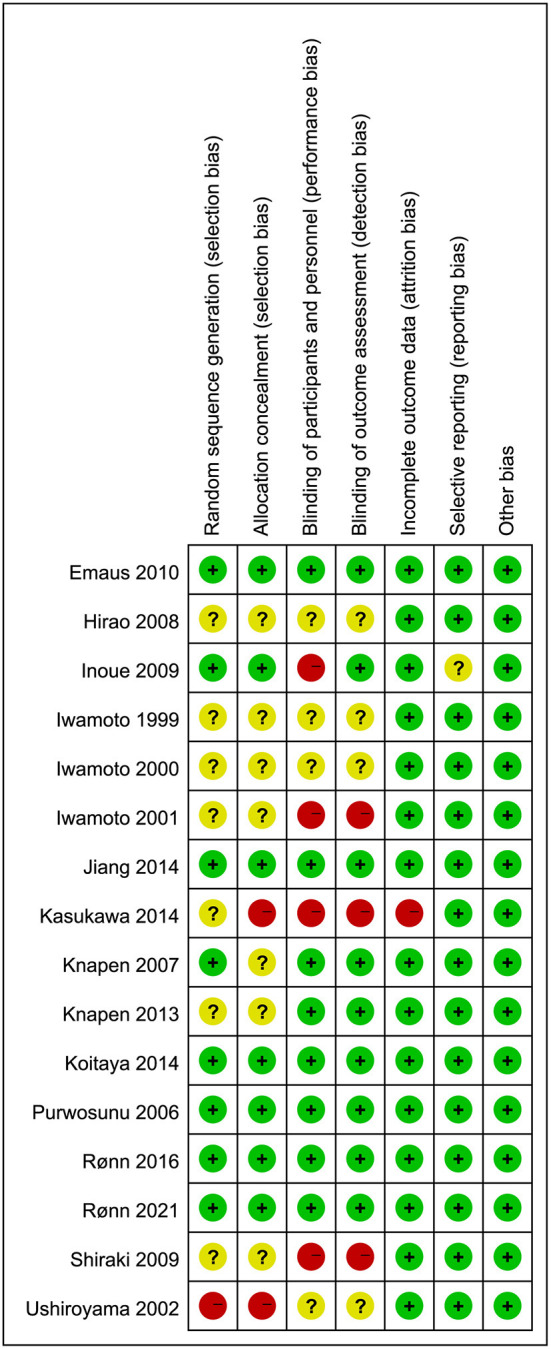
Risk of bias summary of included studies.

Two reviewers (YL. HE, SX.LI) independently assessed the quality of each included randomized controlled trial using the Cochrane Collaboration's tool. Although all studies included in this meta-analysis were randomized controlled trials, some studies did not clearly describe how populations were grouped, resulting in a high risk of selection bias (15, 18, 27, 40, 44–46). There were also some studies that did not implement the double-blind method well, leading to a high risk of performance bias (26, 40, 41, 45). The results of bias risk assessment for the included RCTs are shown in Figure 2.

### Primary outcomes

#### Pooled analysis for the changes in BMD

**Figure 3:**
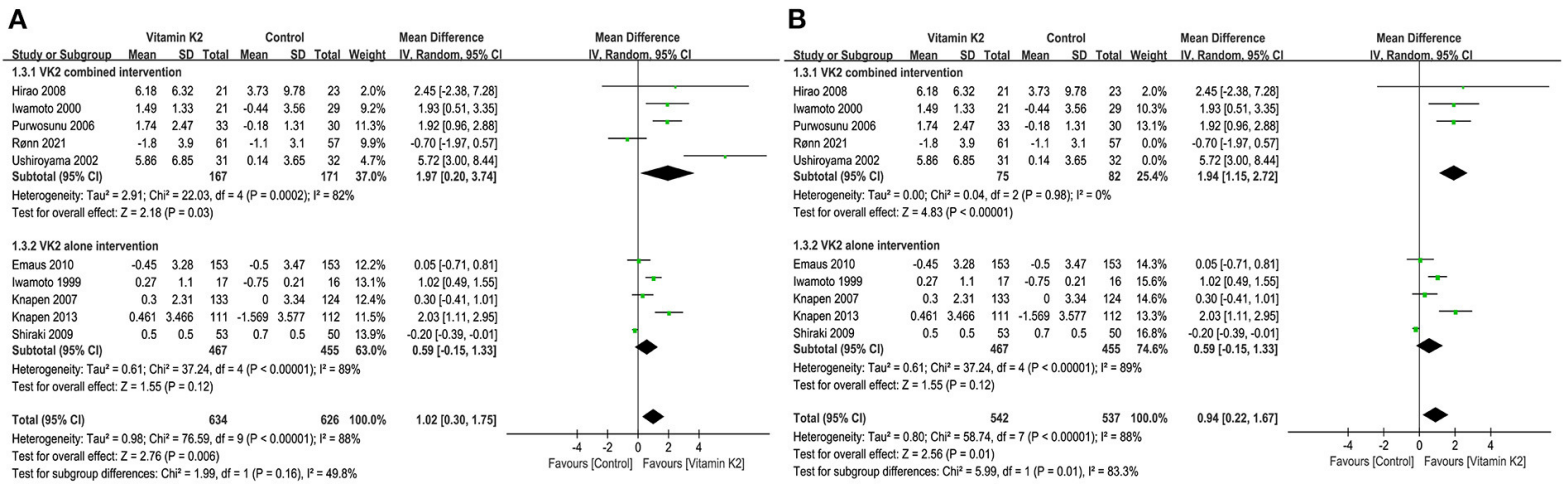
**(A)** Forest plot of the change in BMD LS. **(B)** Forest plot of the change in BMD LS after removed the studies by Rønn and Ushiroyama.

We first analyzed the change in BMD LS before and after treatment with VK2. A total of ten studies (9, 15, 18, 21, 27, 40, 42–44, 46) reported the changes in BMD LS, and the test for the overall effect of these ten studies showed that VK2 maintained and improved BMD LS (MD = 1.02, 95%CI 0.30 to 1.75, P=0.006) compared with the control group (Figure 3A). Subgroup analysis of VK2 combined intervention showed that the effect of VK2 on BMD LS was superior to that of the control group (MD = 1.97, 95% CI 0.20 to 3.74, *P* = 0.03) (Figure 3A). However, the subgroup analysis of VK2 alone intervention showed a similar effect of VK2 and control groups on change in BMD LS (*P* = 0.160) (Figure 3A). In the subgroup of VK2 combined intervention, heterogeneity between studies was eliminated after removing Rønn et al. (9) and Ushiroyama et al. (44) (Figure 3B). And we found that the Z value of the test for overall effect became larger (Z = 4.83), the *P*-value became smaller (*P* < 0.00001), and the 95% confidence interval was narrow down (1.15 to 2.72) (Figure 3B). These changes further suggest that VK2 combined intervention appears to be better at maintaining BMD LS.

**Figure 4:**
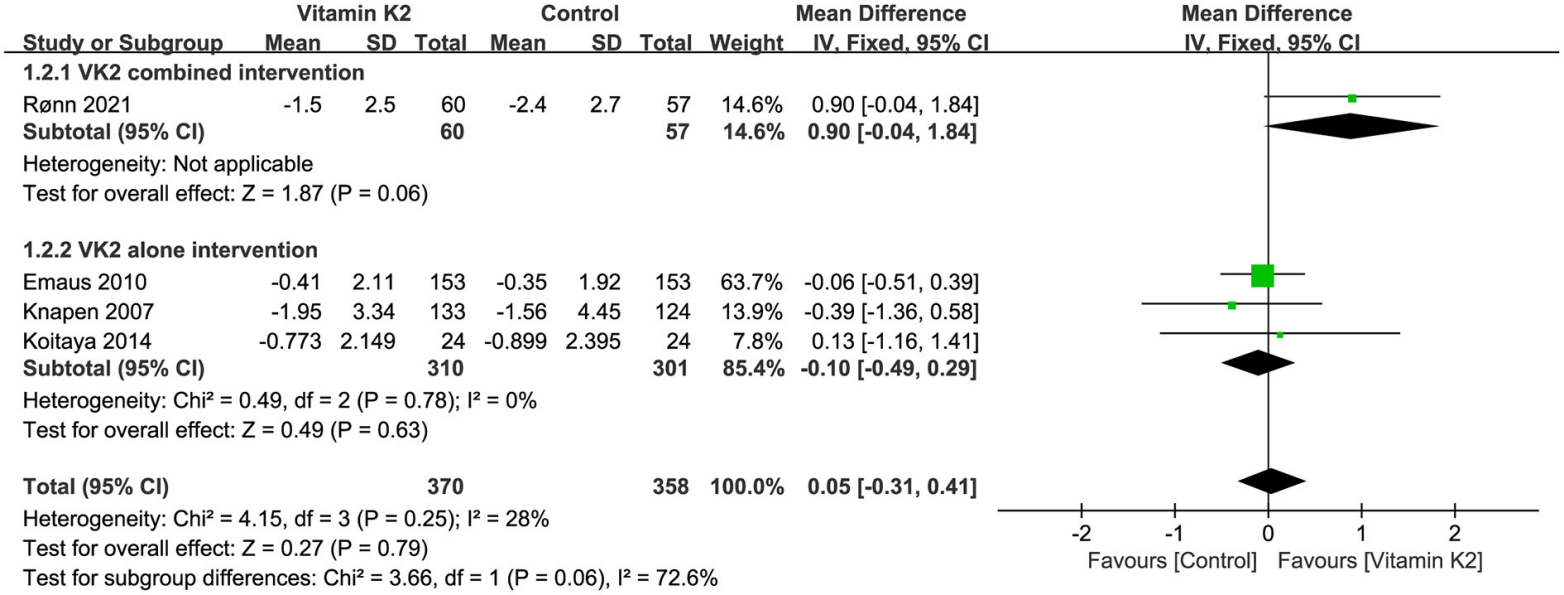
Forest plot of the change in hip BMD.

The changes in hip BMD were reported in four studies (9, 21, 39, 42), and the overall effect test showed no significant difference in hip BMD changes in the VK2 group compared with the control group (*P* = 0.79) (Figure 4).

**Figure 5:**
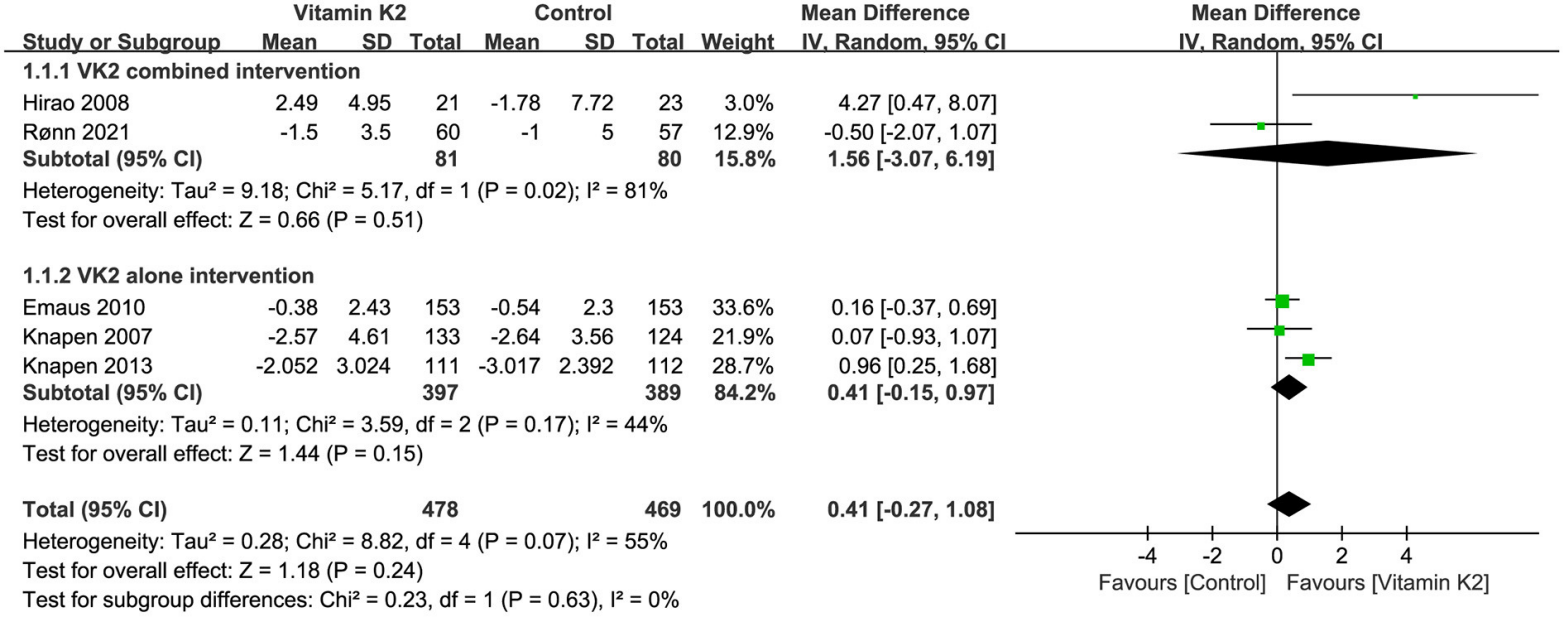
Forest plot of the change in femoral neck BMD.

Five studies (9, 15, 21, 27, 42) reported changes in femoral neck BMD, and the overall effect test showed no significant difference in femoral neck BMD change between the VK2 and control groups (*p* = 0.24) (Figure 5).

**Figure 6:**
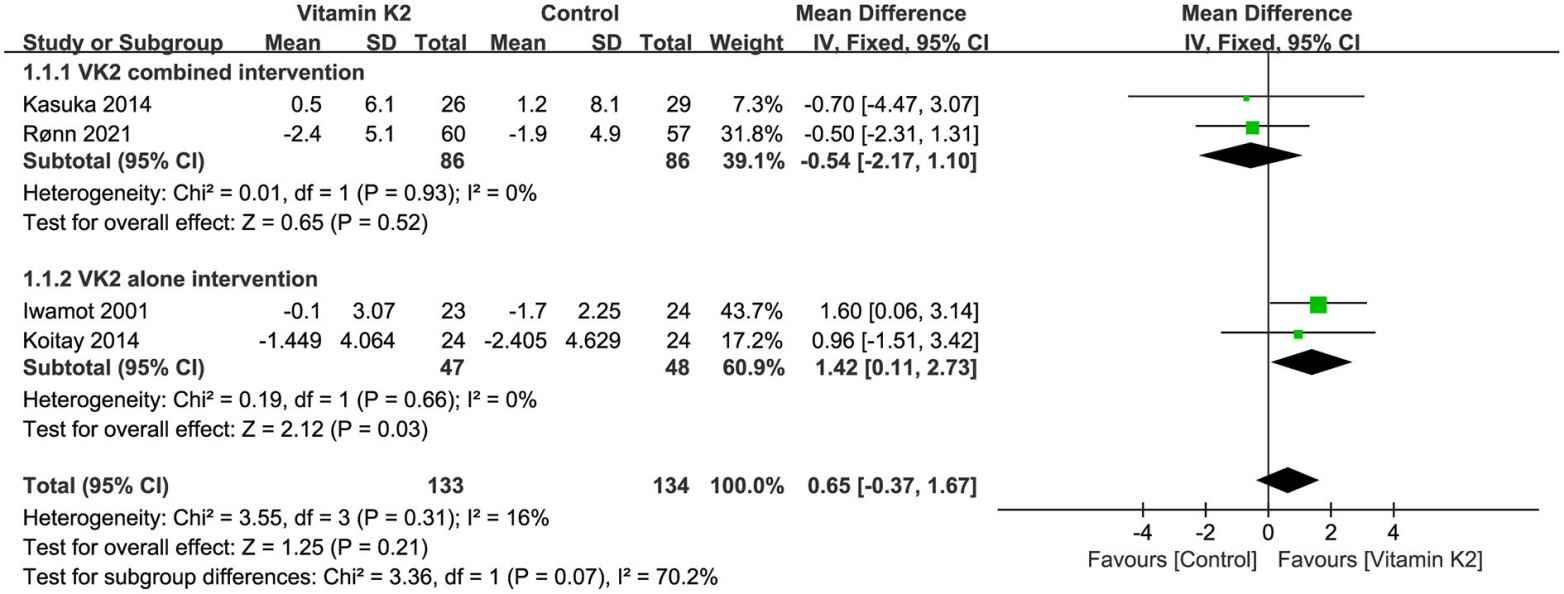
Forest plot of change in forearm BMD.

Four studies (9, 26, 39, 45) reported percentage changes in forearm BMD. Both the overall effect test and the subgroup analysis of VK2 combined intervention showed no significant difference in BMD change between the VK2 and control groups (*P* = 0.21, *P* = 0.52). However, the subgroup analysis of VK2 alone intervention showed that VK2 had a superior effect on forearm BMD than controls (MD = 1.42, 95%CI 0.11 to 2.73, *P* = 0.03) (Figure 6).

### Pooled analysis for fracture incidence

**Figure 7:**
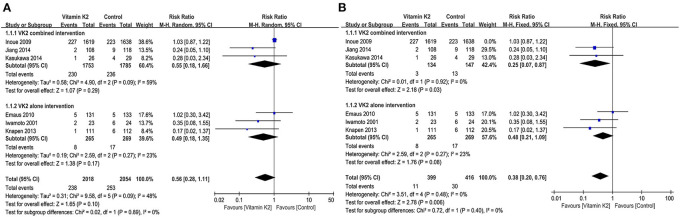
**(A)** Forest plot of the incidence of fractures. **(B)** Forest plot of the incidence of fractures after removed the study by Inoue.

Six studies (14, 15, 21, 26, 41, 45) reported fracture incidence, and the overall effect test showed that VK2 did not reduce the incidence of fractures (RR = 0.56, 95% CI 0.28 to 1.11, *P* = 0.10) (Figure 7A). However, when the study by Inoue et al. (41) was removed, the subgroup analysis of VK2 combined intervention showed a significant difference in fracture incidence between the VK2 and control groups (RR = 0.25, 95% CI 0.07 to 0.87, *P* = 0.03, *I*^2^ = 0%) (Figure 7B). And the overall effect test also showed that VK2 was effective in reducing the incidence of fractures compared with the control group (RR = 0.38, 95% CI 0.20 to 0.76, *P* = 0.006, *I*^2^ = 0%) (Figure 7B).

### Secondary outcomes

#### Pooled analysis for the change in serum uc-OC

**Figure 8:**
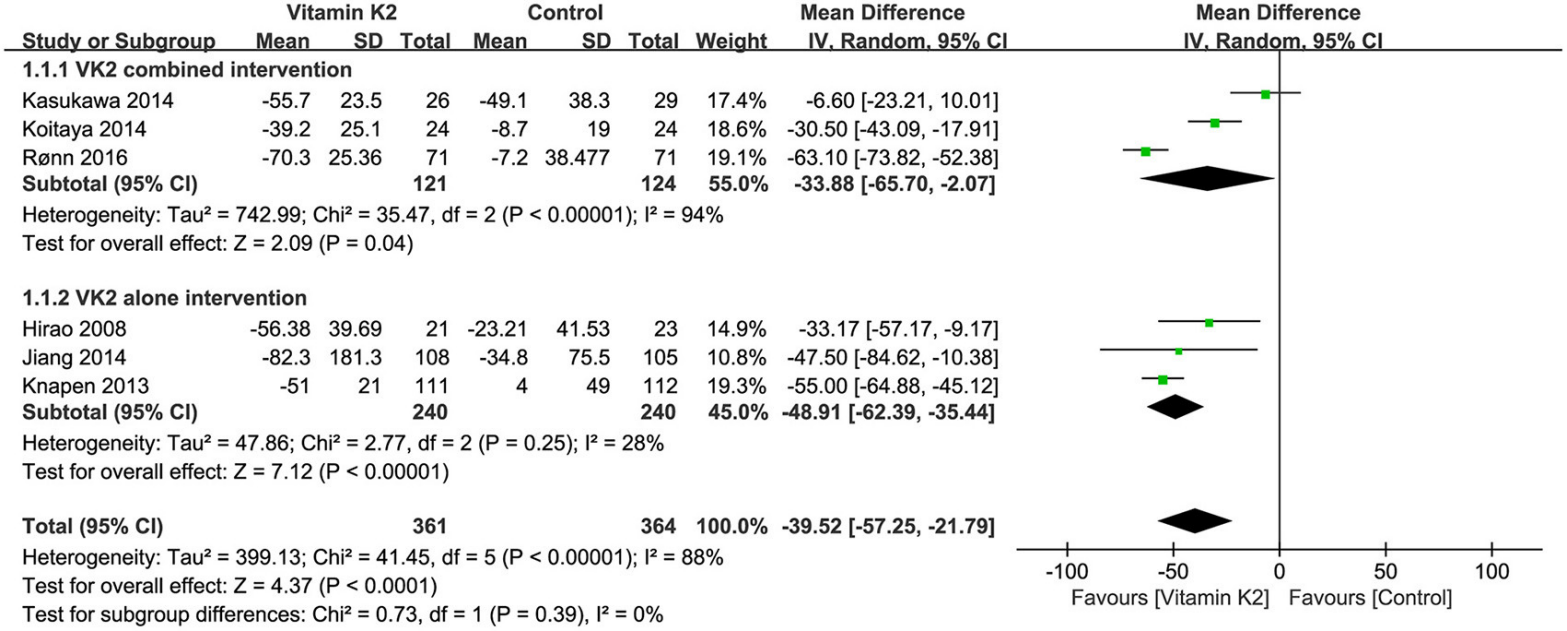
Forest plot of the change in serum uc-OC.

Six studies (14, 15, 26, 27, 38, 39) reported the percentage changes in serum uc-OC. The overall effect test showed a significant difference in serum uc-OC levels between the VK2 and control groups (MD = −39.52, 95%CI−57.25 to−21.79, *P* < 0.0001). Subgroup analyses of both VK2 combined and alone intervention showed that VK2 significantly reduced serum UC-OC levels compared with the control group (*P* < 0.05) (Figure 8).

#### Pooled analysis for the change in serum cOC

**Figure 9:**
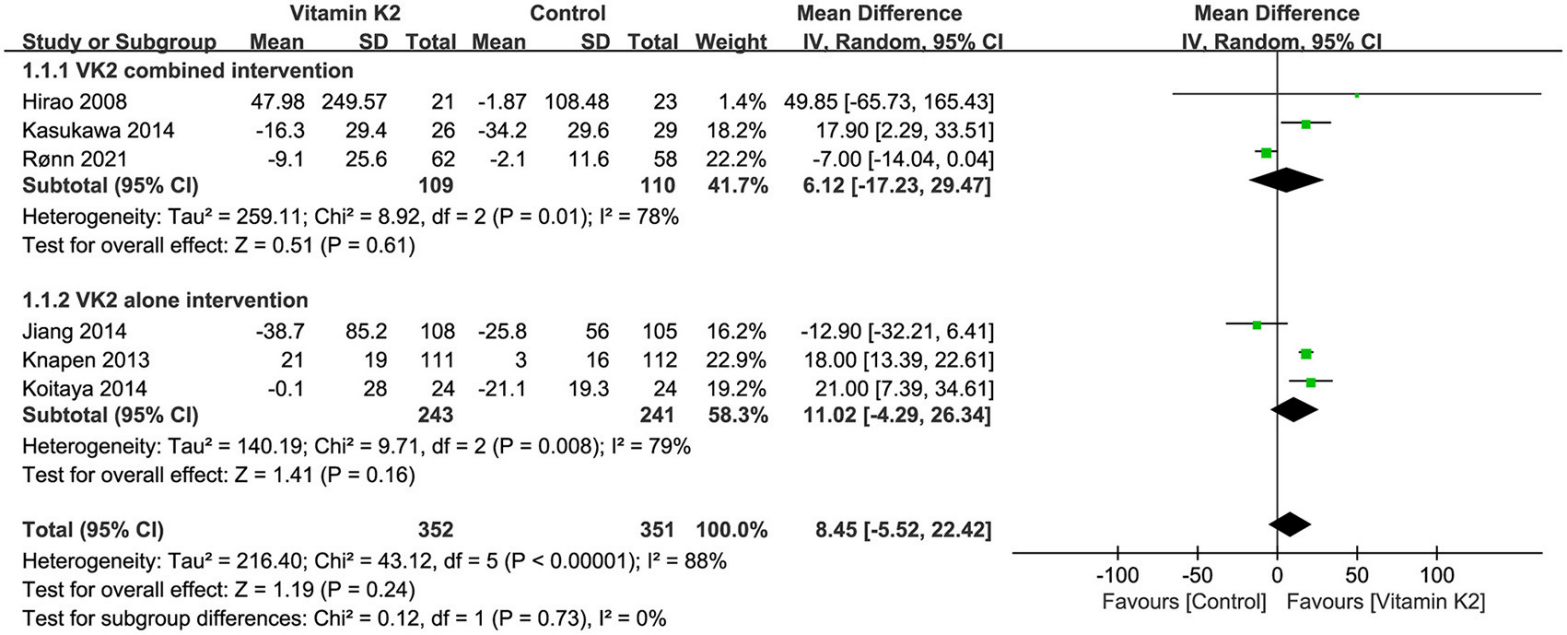
Forest plot of the change in serum cOC.

Six studies (9, 14, 15, 26, 27, 39) reported percentage changes in serum cOC that were not significantly different between the VK2 and control groups by overall effect test (MD = 8.45, 95% CI−5.52 to 22.42, *p* = 0.24) (Figure 9).

#### Pooled analysis for the change in the ratio of serum uc-OC to cOC

**Figure 10:**
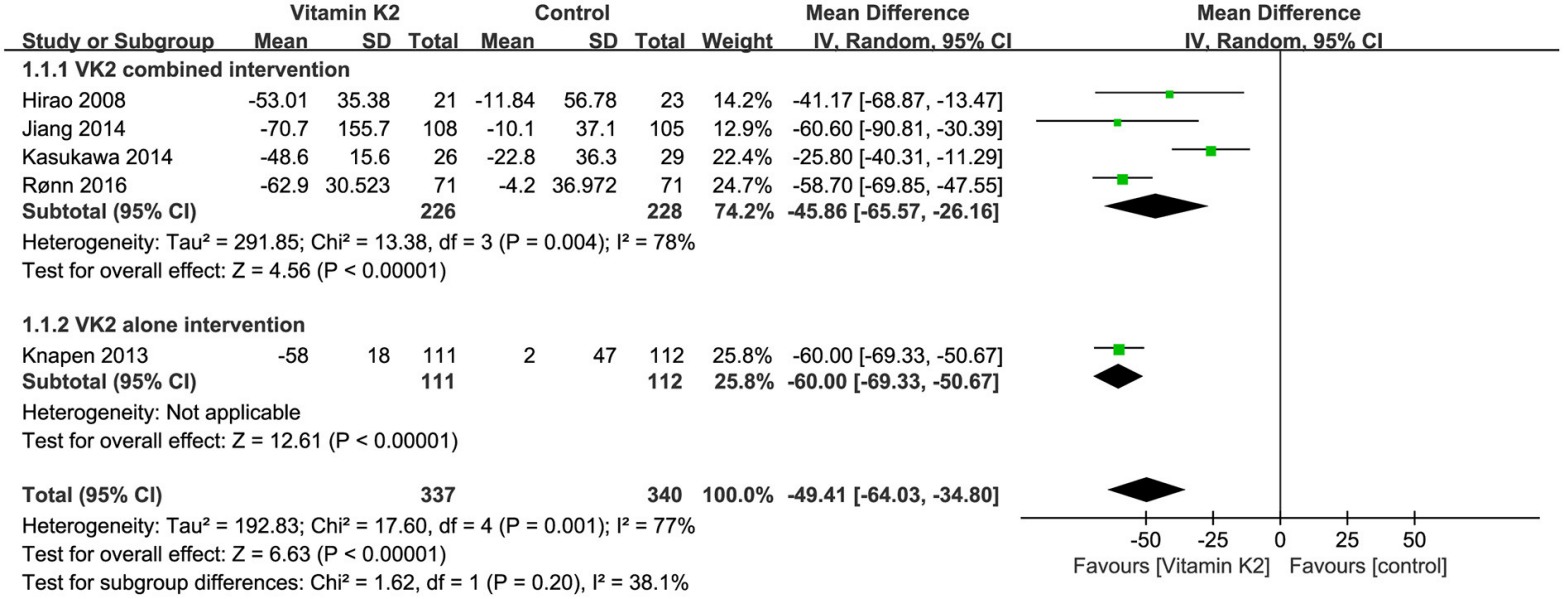
Forest plot of the change in the ratio of serum uc-OC to cOC.

Five studies (14, 15, 26, 27, 38) reported the percentage changes in the ratio of serum uc-OC to cOC, and the overall effect test showed a significant difference between the VK2 and control groups (MD = −49.41, 95% CI 64.03 to−34.80, *P* < 0.00001). Similarly, the subgroup analyses of VK2 combined and alone intervention showed that VK2 significantly reduced the ratio of serum uc-OC to cOC (*P* < 0.00001) (Figure 10).

#### Pooled analysis for the incidence of adverse reactions

**Figure 11:**
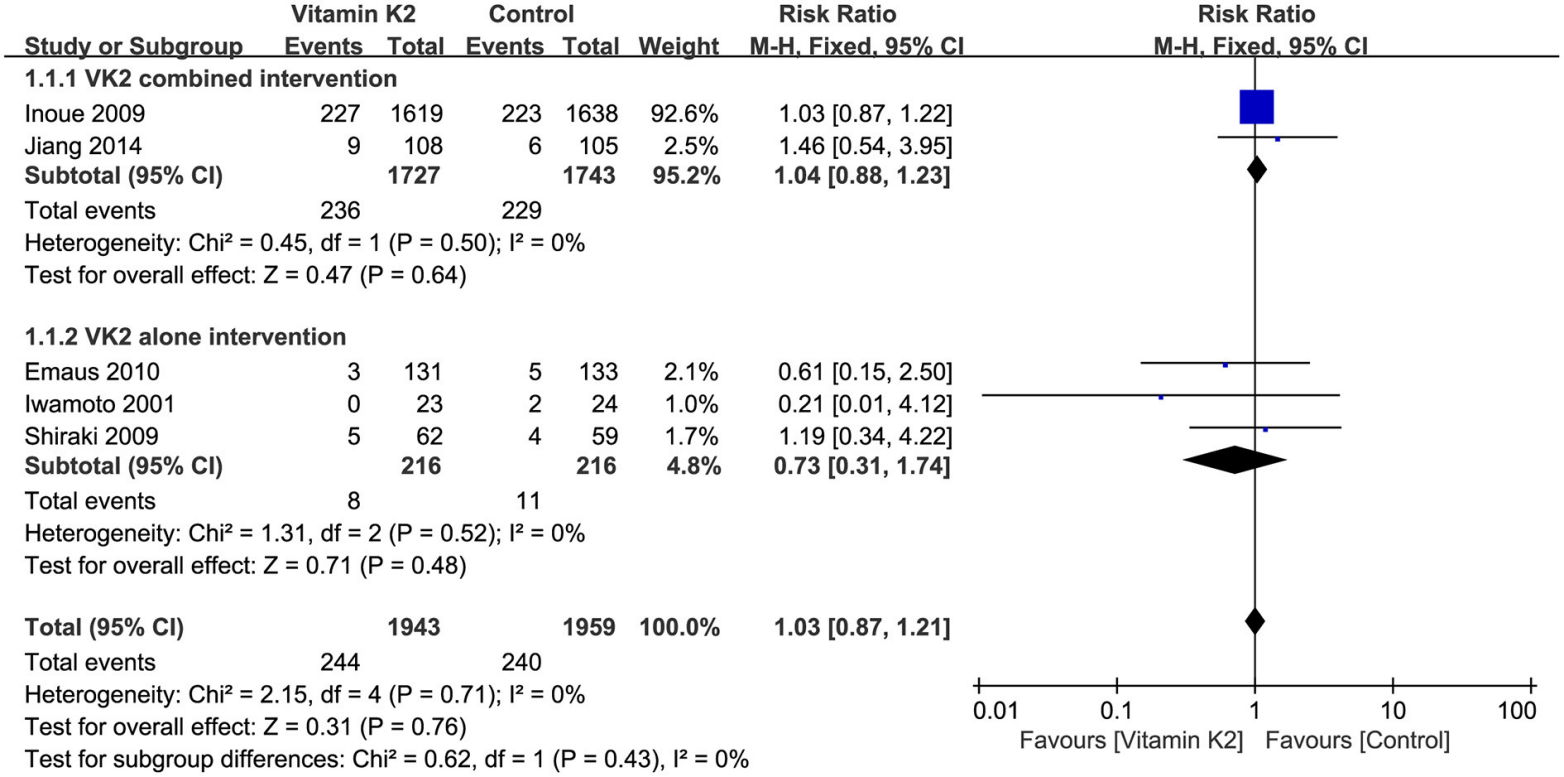
Forest plot of the incidence of adverse reactions.

The incidence of adverse reactions was reported in five studies (14, 21, 40, 41, 45), and overall effect test showed no significant difference in the incidence of adverse reactions between the VK2 and control groups (RR = 1.03, 95% CI 0.87 to 1.21, *P* = 0.76) (Figure 11).

### Publication bias and sensitive analysis

**Figure 12:**
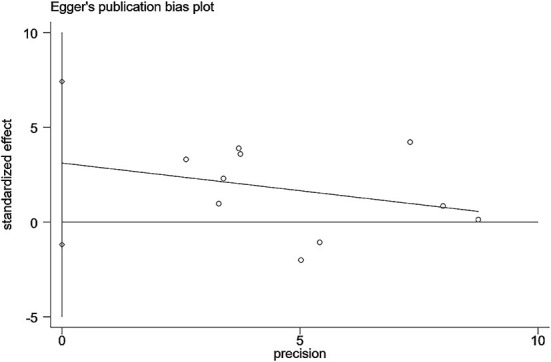
Publication bias test for the overall effect test of BMD LS change.

**Figure 13:**
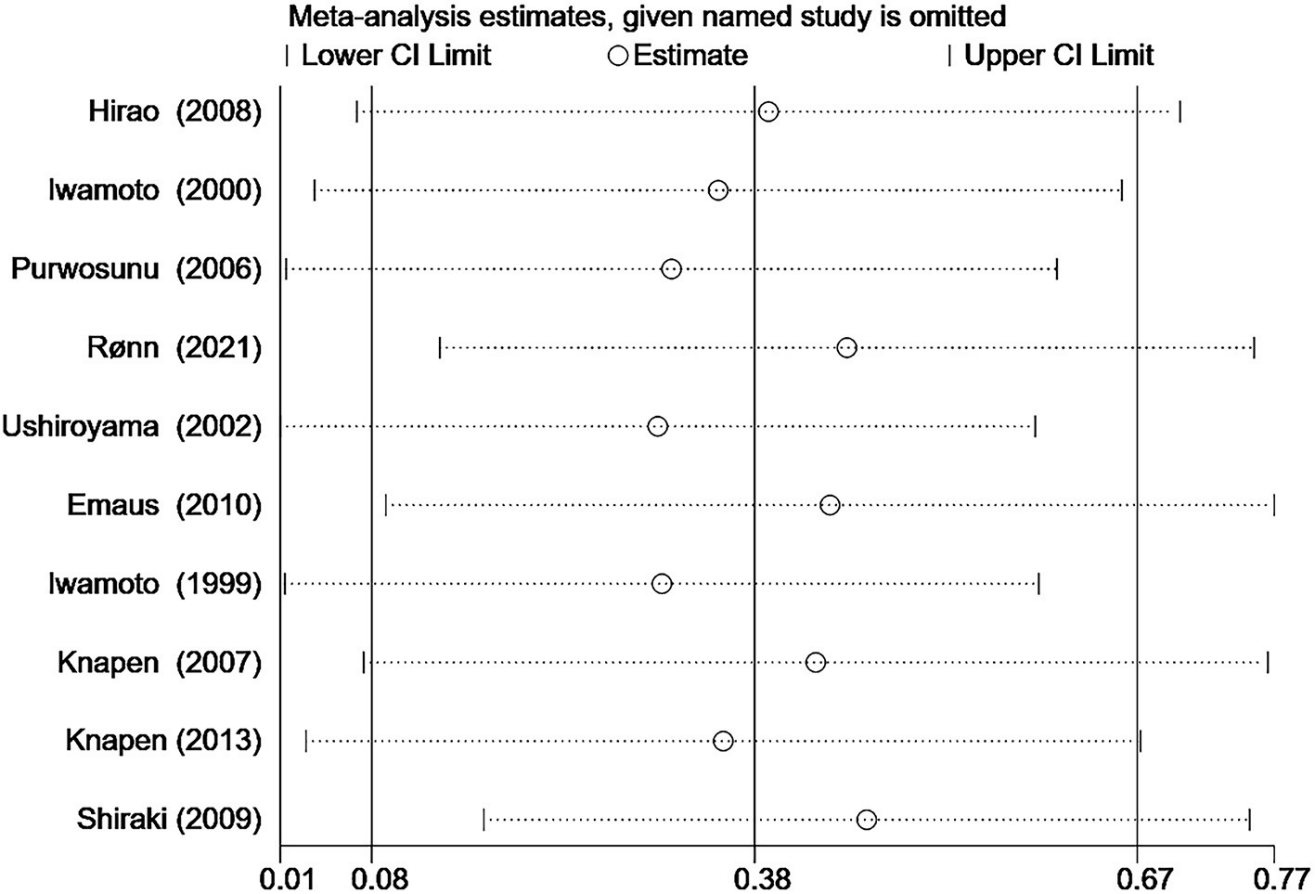
Sensitive analysis for the overall effect test of BMD LS change.

We imported the data from 10 studies (9, 15, 18, 21, 27, 40, 42–44, 46) that reported the percentage changes in BMD LS into STATA 16 software and then assessed publication bias by Egger's quantitative test. The results showed no publication bias for the ten studies (*P* = 0.134, 95%CI−1.19 to 7.41), and each study was roughly symmetrically distributed on both sides of the regression line (Figure 12). In addition, we also performed sensitivity analyses for the 10 studies using STATA 16 software to assess the stability of this meta-analysis model, and the results showed that when any of the 10 studies were removed, the dots representing statistical effect of the remaining studies were distributed around the vertical line of the overall statistical effect and within 95% confidence interval (Figure 13).

## Discussion

We searched for studies according to the PRISMA statement and screened studies according to the inclusion and exclusion criteria, and ultimately 16 RCTs were included in this meta-analysis. By pooled analysis, we found that VK2 maintained and improved the BMD LS, and the subgroup analysis of VK2 combined intervention yielded the same conclusion, but the subgroup analysis of VK2 intervention alone did not show the difference compared with the control group. In addition, the results of this meta-analysis showed no significant differences between the VK2 and control groups in terms of BMD changes in the hip, femoral neck and forearm. The overall effect test for fracture incidence showed that VK2 reduced fracture incidence after removing study with large heterogeneity (RR = 0.38,95% CI 0.20 to 0.76, *P* = 0.006, *I*^2^ = 0%) (41).

It is well known that the main aim of prevention and treatment of OP is to prevent fractures, so we took the BMD changes and fracture incidence as the primary outcome indicators in this meta-analysis. However, of all RCTs included in this meta-analysis, only 6 studies (14, 15, 21, 26, 41, 45) reported the incidence of fractures. Therefore, the effect of VK2 on fracture incidence cannot be assessed very accurately in this meta-analysis. The pooled analysis of the 6 studies showed that VK2 cannot reduce the fracture incidence, and subgroup analyses of VK2 combined and alone intervention also showed the similar results. However, when we performed sensitivity analyses on these six studies, we found that the study by Inoue et al. (41) was relatively heterogeneous in comparison with other studies. When the study by Inoue et al. was removed, the result of this meta-analysis showed that VK2 reduced the incidence of fractures. But in fact, the exclusion of the study by Inoue et al. may cause selection bias, because the sample size of this study was very large and the follow-up period was longer (48 months) than other studies. In the study by Inoue et al. there was no significant difference in the incidence of fractures between the VK2 and control groups. However, this study showed a significant reduction in the incidence of new vertebral fractures in patients treated with VK2 who had already had vertebral fractures, and it also showed that VK2 had a positive effect on the elderly population suffering from severe bone loss. Therefore, although the heterogeneity of this study is relatively large, it is undeniably a very valuable study. In view of this, we need to be cautious in excluding this study and also cautious in drawing conclusion that VK2 can reduce the incidence of fractures.

Osteocalcin (OC), also known as a gamma-carboxyl bone glaprotein (BGP), is a vitamin K-dependent protein that is synthesized and secreted by osteoblasts (47). OC has biological effects in regulating bone metabolism after OC is γ -carboxylated, but the process requires sufficient VK. Depending on the degree of carboxylation of osteocalcin, osteocalcin includes fully carboxylated osteocalcin (cOC) and undercarboxylated osteocalcin (uc-OC), while cOC can bind to hydroxyapatite and thus promote calcium deposition in bones. When serum VK is deficient, the carboxylation ability of osteocalcin will be weakened, serum uc-OC levels will be increased, and cOC levels will be decreased (48, 49). And low serum VK levels and high uc-OC levels are considered as risk factors for hip fracture in older women (50). In this meta-analysis, the overall effect test showed that VK2 significantly reduced serum uc-OC levels and the ratio of serum uc-OC to cOC, while subgroup analyses of the VK2 combined intervention and alone intervention also yielded similar conclusions. However, there was no significant difference in serum cOC levels between the VK2 group and the control group. Theoretically, when VK2 is maintained at a relatively high levels in serum, the γ-carboxylation of OC would be enhanced, thus contributing to the increase in serum cOC levels and the decrease in uc-OC levels. It should not be forgotten, however, that with increasing serum VK2 levels, more and more OC can become carboxylated and functional. And more of the functional OC can be bound to the bones rather than circulating in the blood, so serum cOC levels may not increase significantly.

Five studies (14, 21, 40, 41, 45) reported the incidence of adverse reactions in evaluating the safety of VK2 in the prevention and treatment of PMOP. The pooled analysis of the five studies showed that VK2 did not increase the incidence of adverse reactions compared to the controls (RR = 1.03, 95% CI 0.87 to 1.21, *P* = 0.76). It suggests to us that the use of VK2 for the prevention and treatment of PMOP is safe in the present situation.

In conclusion, we may conclude that VK2 is effective in the prevention and treatment of PMOP, because VK2 appears to maintain and improve the BMD LS. And VK2 may be particularly effective when used in combination with other preventive or therapeutic measures, such as vitamin D, calcium, or alendronate. The results of this meta-analysis gave us the greatest confidence that VK2 can significantly reduce serum levels of uc-OC. In the effect of VK2, more OC undergoes γ-carboxylation and are converted to cOC, which can promote bone mineralization and increase bone strength. Meanwhile this meta-analysis also suggests that VK2 may reduce the incidence of fractures, but this conclusion needs to be further verified by more multi-center, large sample size and prospective studies.

Although the 16 studies included in this meta-analysis were all randomized controlled trials, the following problems still remained in our meta-analysis: (1) the quality of the included studies was uneven, and there were various biases (selection bias, performance bias, detection bias, etc.); (2) the sample sizes of some RCTs were too small, which might lead to the conclusions that were accidental; (3) the follow-up time of the included studies was different, and we did not perform more detailed groupings; (4) the majority of the included studies were conducted in Japan, and these studies that concluded that VK2 had a positive effect on prevention and treatment of PMOP were also primarily conducted in Japan. Therefore, it remains to be discussed whether the positive effect of VK2 on prevention and treatment of PMOP can be extended to other countries.

## Data availability statement

The original contributions presented in the study are included in the article/Supplementary material, further inquiries can be directed to the corresponding authors.

## Author contributions

M-lM, Z-jM, and HS carried out the study design, study selection, data extraction, statistical analysis, and drafted the manuscript. BY, B-jR, and W-dZ participated in the study selection, data extraction, and drafted the manuscript. Y-lH and S-xL evaluated the quality of included studies. HD and Y-xW participated in the discussion for any discrepancies and supervised the study. All authors read and approved the final manuscript.

## Funding

This study was supported by grants from TCM Science and Technology Development Program Project of Jiangsu Province (ZT202117) and Yangzhou Key Laboratory Cultivation Project (YZ2021143).

## Conflict of interest

The authors declare that the research was conducted in the absence of any commercial or financial relationships that could be construed as a potential conflict of interest.

## Publisher's note

All claims expressed in this article are solely those of the authors and do not necessarily represent those of their affiliated organizations, or those of the publisher, the editors and the reviewers. Any product that may be evaluated in this article, or claim that may be made by its manufacturer, is not guaranteed or endorsed by the publisher.
